# A co-crystal of 1,10-phenanthroline with boric acid: a novel aza-aromatic complex

**DOI:** 10.1107/S1600536813015134

**Published:** 2013-06-12

**Authors:** Arsalan Mirjafari, Lam Pham, Philip J. Smith, Richard E. Sykora, James H. Davis

**Affiliations:** a Florida Gulf Coast University, Department of Chemistry and Mathematics, Fort Myers, FL 33965-6565, USA; b University of South Alabama, Department of Chemistry, Mobile, AL 36688-0002

## Abstract

The title compound, C_12_H_8_N_2_·2B(OH)_3_, is best described as a host–guest complex in which the B(OH)_3_ mol­ecules form a hydrogen-bonded cyclic network of layers parallel to the *ab* plane into which the 1,10-phenanthroline mol­ecules are bound. An extensive network of hydrogen bonds are responsible for the crystal stability. No π-stacking inter­actions occur between the 1,10-phenanthroline mol­ecules.

## Related literature

For the design and synthesis of novel systems of non-covalent hosts involving hydrogen bonds, see: Pedireddi *et al.* (1997 ▶). In the field of supermolecular synthesis, recognition between the complementary functional groups is a main factor for the evaluation of influence of noncovalent inter­actions in the formation of specific architecture, see: Lehn (1990 ▶). The ability of the –B(OH)_2_ functionality to form a variety of hydrogen bonds through different conformations makes it a very suitable moiety for the synthesis of novel mol­ecular complexes, see: Lee *et al.* (2005 ▶). It is known to have an affinity for pyridyl N atoms, often forming O—H⋯N hydrogen bonds, as observed in some crystals of boronic acids with aza compounds (Talwelkar & Pedireddi, 2010 ▶). Non-covalent hosts are generally designed and synthesized by employing appropriate functional groups at required symmetry positions to form a cyclic network through the hydrogen bonds, see: Pedireddi (2001 ▶). This effect has been observed in simple mol­ecular adducts such as 1,10-phenanthroline and water (Tian *et al.*, 1995 ▶).
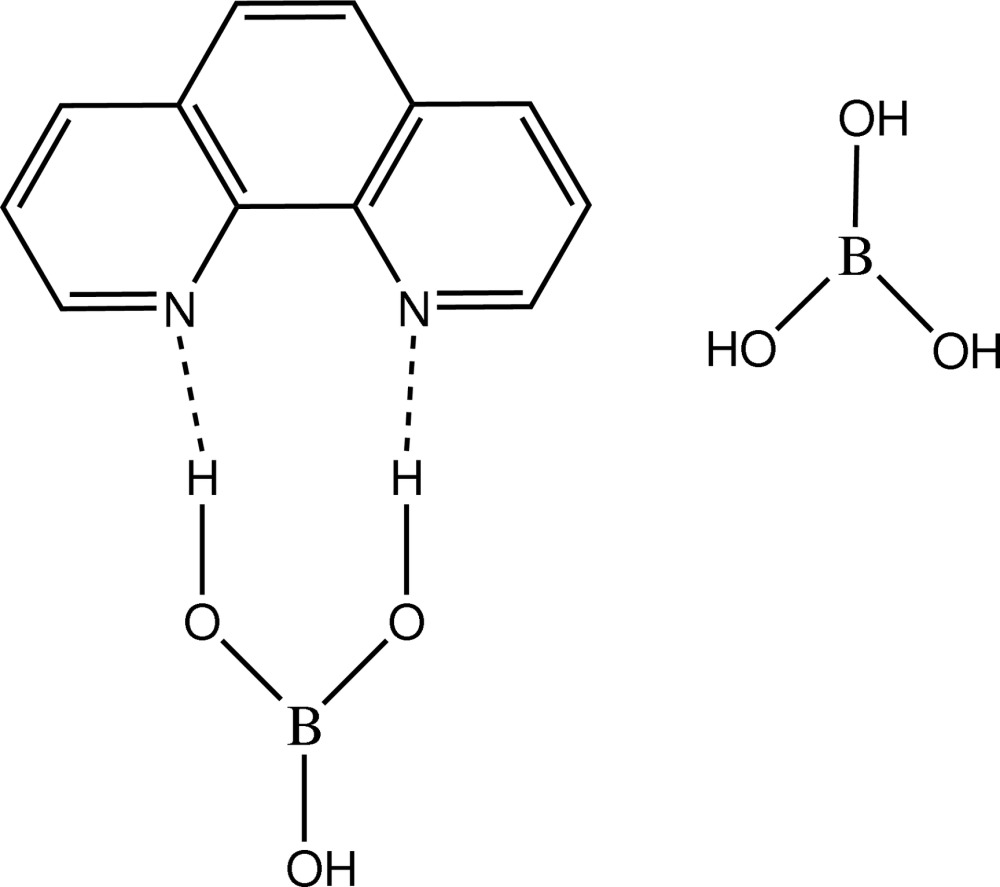


## Experimental

### 

#### Crystal data

C_12_H_8_N_2_·2BH_3_O_3_
*M* *_r_* = 303.87
Triclinic, 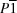

*a* = 7.1390 (13) Å
*b* = 9.6189 (13) Å
*c* = 10.4756 (15) Å
α = 93.767 (11)°
β = 101.546 (14)°
γ = 90.644 (13)°
*V* = 703.05 (19) Å^3^
*Z* = 2
Mo *K*α radiation
μ = 0.11 mm^−1^
*T* = 295 K
0.35 × 0.16 × 0.09 mm

#### Data collection

Oxford Diffraction Xcalibur Eos diffractometer
Absorption correction: multi-scan [*CrysAlis PRO* (Oxford Diffraction, 2011 ▶) based on Clark & Reid (1995 ▶)] *T*_min_ = 0.956, *T*_max_ = 1.000
10473 measured reflections
2580 independent reflections
1972 reflections with *I* > 2σ(*I*)
*R*_int_ = 0.023

#### Refinement

*R*[*F* ^2^ > 2σ(*F* ^2^)] = 0.036
*wR*(*F* ^2^) = 0.096
*S* = 1.02
2580 reflections
199 parameters
H-atom parameters constrained
Δρ_max_ = 0.17 e Å^−3^
Δρ_min_ = −0.13 e Å^−3^

### 

Data collection: *CrysAlis PRO* (Oxford Diffraction, 2011 ▶); cell refinement: *CrysAlis PRO*; data reduction: *CrysAlis PRO*; program(s) used to solve structure: *SHELXS97* (Sheldrick, 2008 ▶); program(s) used to refine structure: *SHELXL97* (Sheldrick, 2008 ▶); molecular graphics: *SHELXTL* (Sheldrick, 2008 ▶) and *OLEX2* (Dolomanov *et al.*, 2009 ▶); software used to prepare material for publication: *publCIF* (Westrip, 2010) ▶.

## Supplementary Material

Crystal structure: contains datablock(s) I, New_Global_Publ_Block. DOI: 10.1107/S1600536813015134/ez2287sup1.cif

Structure factors: contains datablock(s) I. DOI: 10.1107/S1600536813015134/ez2287Isup2.hkl

Supplementary material file. DOI: 10.1107/S1600536813015134/ez2287Isup3.cml

Additional supplementary materials:  crystallographic information; 3D view; checkCIF report

## Figures and Tables

**Table 1:** Hydrogen-bond geometry (Å, °)

*D*—H⋯*A*	*D*—H	H⋯*A*	*D*⋯*A*	*D*—H⋯*A*
O1—H1⋯N2	0.85	1.90	2.7360 (16)	169
O2—H2⋯N1	0.85	1.88	2.7132 (17)	167
O3—H3⋯O1^i^	0.85	1.86	2.7076 (15)	177
O4—H4⋯O3^i^	0.85	1.89	2.7286 (16)	16
O5—H5⋯O4^ii^	0.85	1.89	2.7355 (18)	179
O6—H6⋯O2^iii^	0.85	1.95	2.7946 (17)	172

Symmetry codes: (i) 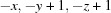; (ii) 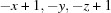; (iii) 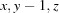.

**Fig. 1.**
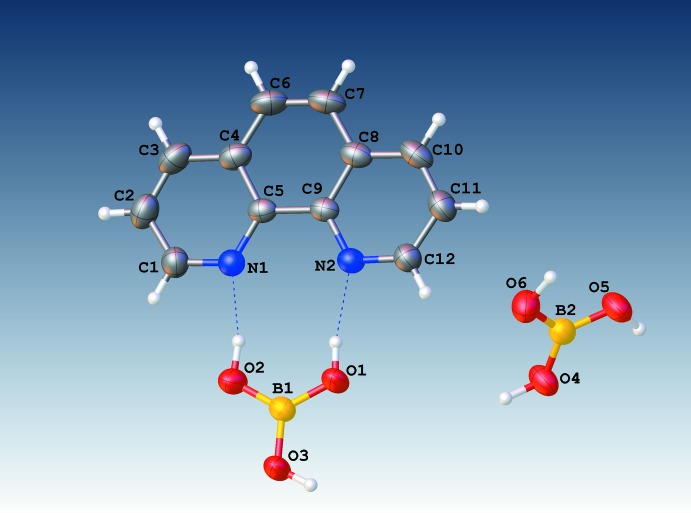
The molecular structure of I, with the atom-numbering scheme. Displacement ellipsoids for non-hydrogen atoms are drawn at the 50% probability level.

**Fig. 2.**
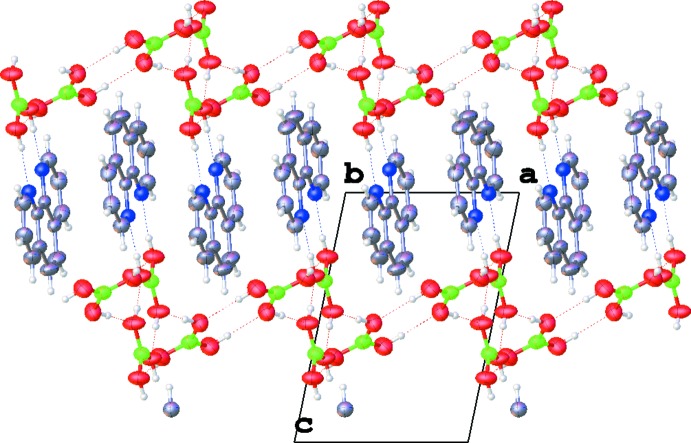
A packing diagram of I viewed along the b axis.

**Fig. 3.**
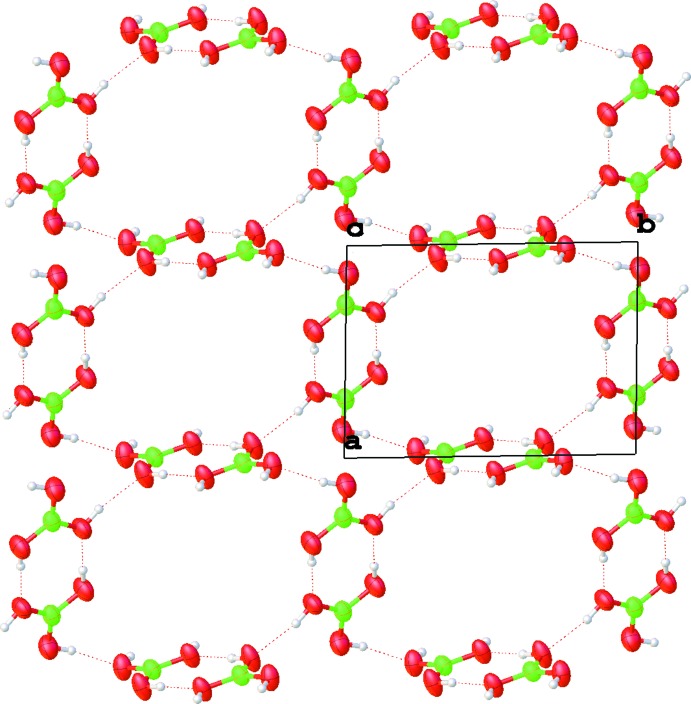
A representation of the two-dimensional B(OH)3 layers formed via hydrogen bonding in the structure of I.

## supplementary crystallographic information

### Comment

The design and synthesis of novel systems of noncovalent hosts involving hydrogen bonds is a vast research area in both molecular and supermolecular chemistry, see Pedireddi *et al.* (1997). In the field of supermolecular synthesis, recognition between the complementary functional groups is a main factor for the evaluation of influence of noncovalent interaction in the formation of specific architecture, see: Lehn (1990). In recent times, boric acid derivatives have been well considered to be potential co-crystal formers. In fact, the ability of –B(OH)_2_ functionality to form a variety of hydrogen bonds through different conformations makes it a very suitable moiety for the synthesis of novel molecular complexes, see Lee *et al.* (2005). The –B(OH)_2_ moiety is known to have an affinity for pyridyl N-atoms, often forming O—H···N hydrogen bonds, as observed in some crystals of boronic acids with aza compounds,see Talwelkar & Pedireddi (2010).

Non-covalent hosts are generally designed and synthesized by employing appropriate functional groups at required symmetry positions to form a cyclic network through the hydrogen bonds, see Pedireddi (2001). This effect has been observed vividly in simple molecular adduct such as 1,10-phenanthroline and water, see Tian *et al.* (1995). In this complex, a water molecule interacts with a molecule of 1,10-phenanthroline through O–H···N hydrogen bonds and an unique aza-aromatic complex is formed. In the latter, 1,10-phenanthroline could be considered as a host. Herein, we report the crystal structure of boric acid with 1,10-phenanthroline as an aza-donor compound.

As seen in Figure 1, the phen molecule forms a H-bonded adduct *via* two B–O–H···N interacts from one of the included B(OH)_3_ moieties. A strong network of hydrogen bonds among the B(OH)_3_ units forms a layered structure with alternating B(OH)_3_ and phen layers that reside in the *ab* planes (Figure 2). The B(OH)_3_ layers alone can be described as a cyclic network formed by hydrogen bonding interactions as can be seen in Figure 3. There are not any significant π-stacking interactions between the phen molecules.

### Experimental

(CH_3_)_3_NBH_3_ (0.73 g, 10 mmol) and iodine (2.54 g, 5 mmol) were dissolved in toluene (4 ml) and stirred for 30 min. A solution of 1,10-phenanthroline (1.98 g, 10 mmol) in toluene (4 ml) was added, and the mixture refluxed overnight. The solution was cooled to room temperature, during which process orange-brown crystals were formed. The product was recrystallized twice from CH_3_CN to obtain analytically pure, red-brown crystalline product.

^1^H NMR (DMSO-*d_6_*, 300 MHz): *δ*_H_ 9.22 (dd, *J* = 2.8, 1.6 Hz, 2H), 8.67 (dd, *J* = 6.3, 1.6 Hz, 2H), 8.14 (s, 2H), 7.93 (q, *J* = 4.4 Hz, 2H), 6.62 (br, 2H); ^13^C NMR (DMSO-*d_6_*, 100 MHz): *δ*_C_ 151.67, 146.27, 139.09, 130.58, 128.77, 125.66.

### Refinement

H-atoms were placed in calculated positions and allowed to ride during subsequent refinement, with *U*_iso_(H) = 1.2*U*_eq_(C) and C—H distances of 0.93 Å for the aromatic H atoms and with *U*_iso_(H) = 1.5*U*_eq_(C) and O—H distances of 0.85 Å for hydroxyl H atoms.

### Crystal data

**Table:**

C_12_H_8_N_2_·2BH_3_O_3_	*Z* = 2
*M**_r_* = 303.87	*F*(000) = 316
Triclinic, *P*1	*D*_x_ = 1.435 Mg m^−^^3^
Hall symbol: -P 1	Mo *K*α radiation, λ = 0.71073 Å
*a* = 7.1390 (13) Å	Cell parameters from 3335 reflections
*b* = 9.6189 (13) Å	θ = 3.2–25.3°
*c* = 10.4756 (15) Å	µ = 0.11 mm^−^^1^
α = 93.767 (11)°	*T* = 295 K
β = 101.546 (14)°	Prism, brown
γ = 90.644 (13)°	0.35 × 0.16 × 0.09 mm
*V* = 703.05 (19) Å^3^	

### Data collection

**Table:**

Oxford Diffraction Xcalibur Eos diffractometer	2580 independent reflections
Radiation source: fine-focus sealed tube	1972 reflections with *I* > 2σ(*I*)
Graphite monochromator	*R*_int_ = 0.023
Detector resolution: 16.0514 pixels mm^-1^	θ_max_ = 25.4°, θ_min_ = 3.2°
ω scans	*h* = −8→8
Absorption correction: multi-scan [*CrysAlis PRO* (Oxford Diffraction, 2011) based on Clark & Reid (1995)]	*k* = −11→11
*T*_min_ = 0.956, *T*_max_ = 1.000	*l* = −12→12
10473 measured reflections	

### Refinement

**Table:**

Refinement on *F*^2^	Primary atom site location: structure-invariant direct methods
Least-squares matrix: full	Secondary atom site location: difference Fourier map
*R*[*F*^2^ > 2σ(*F*^2^)] = 0.036	Hydrogen site location: inferred from neighbouring sites
*wR*(*F*^2^) = 0.096	H-atom parameters constrained
*S* = 1.02	*w* = 1/[σ^2^(*F*_o_^2^) + (0.044*P*)^2^ + 0.1204*P*] where *P* = (*F*_o_^2^ + 2*F*_c_^2^)/3
2580 reflections	(Δ/σ)_max_ < 0.001
199 parameters	Δρ_max_ = 0.17 e Å^−^^3^
0 restraints	Δρ_min_ = −0.13 e Å^−^^3^

### Special details

**Table:**

Geometry. All e.s.d.'s (except the e.s.d. in the dihedral angle between two l.s. planes) are estimated using the full covariance matrix. The cell e.s.d.'s are taken into account individually in the estimation of e.s.d.'s in distances, angles and torsion angles; correlations between e.s.d.'s in cell parameters are only used when they are defined by crystal symmetry. An approximate (isotropic) treatment of cell e.s.d.'s is used for estimating e.s.d.'s involving l.s. planes.
Refinement. Refinement of *F*^2^ against ALL reflections. The weighted *R*-factor *wR* and goodness of fit *S* are based on *F*^2^, conventional *R*-factors *R* are based on *F*, with *F* set to zero for negative *F*^2^. The threshold expression of *F*^2^ > 2σ(*F*^2^) is used only for calculating *R*-factors(gt) *etc*. and is not relevant to the choice of reflections for refinement. *R*-factors based on *F*^2^ are statistically about twice as large as those based on *F*, and *R*- factors based on ALL data will be even larger.

### Fractional atomic coordinates and isotropic or equivalent isotropic displacement parameters (Å^2^)

**Table:**

	*x*	*y*	*z*	*U*_iso_*/*U*_eq_	
B1	0.0170 (3)	0.65229 (18)	0.64598 (18)	0.0421 (4)	
B2	0.2786 (3)	−0.00132 (19)	0.59856 (18)	0.0439 (4)	
C1	0.1542 (2)	0.83587 (17)	1.04155 (17)	0.0488 (4)	
H1A	0.1058	0.9001	0.9816	0.059*	
C2	0.1999 (3)	0.8822 (2)	1.17305 (19)	0.0573 (5)	
H2A	0.1815	0.9743	1.1999	0.069*	
C3	0.2718 (3)	0.7893 (2)	1.26068 (18)	0.0574 (5)	
H3A	0.3014	0.8168	1.3493	0.069*	
C4	0.3018 (2)	0.65145 (18)	1.21789 (15)	0.0464 (4)	
C5	0.2496 (2)	0.61314 (16)	1.08273 (14)	0.0369 (4)	
C6	0.3852 (3)	0.5520 (2)	1.30610 (17)	0.0587 (5)	
H6A	0.4195	0.5784	1.3948	0.070*	
C7	0.4149 (3)	0.4217 (2)	1.26368 (18)	0.0567 (5)	
H7	0.4714	0.3593	1.3231	0.068*	
C8	0.3612 (2)	0.37654 (17)	1.12795 (16)	0.0434 (4)	
C9	0.2778 (2)	0.47095 (15)	1.03670 (14)	0.0361 (3)	
C10	0.3890 (2)	0.23972 (17)	1.08186 (18)	0.0521 (5)	
H10	0.4451	0.1755	1.1396	0.062*	
C11	0.3337 (2)	0.20134 (17)	0.95254 (18)	0.0513 (4)	
H11	0.3496	0.1107	0.9206	0.062*	
C12	0.2522 (2)	0.30118 (16)	0.86861 (16)	0.0449 (4)	
H12	0.2150	0.2741	0.7802	0.054*	
N1	0.17492 (17)	0.70652 (13)	0.99611 (12)	0.0403 (3)	
N2	0.22495 (17)	0.43158 (12)	0.90711 (12)	0.0385 (3)	
O1	0.07961 (17)	0.52138 (11)	0.66548 (10)	0.0515 (3)	
H1	0.1261	0.5050	0.7441	0.077*	
O2	0.02929 (18)	0.75343 (11)	0.74334 (11)	0.0548 (3)	
H2	0.0796	0.7268	0.8180	0.082*	
O3	−0.06394 (19)	0.68679 (11)	0.52361 (11)	0.0583 (4)	
H3	−0.0642	0.6217	0.4646	0.087*	
O4	0.31472 (16)	0.10158 (11)	0.52210 (12)	0.0539 (3)	
H4	0.2251	0.1595	0.5081	0.081*	
O5	0.39825 (18)	−0.11040 (12)	0.61562 (12)	0.0587 (3)	
H5	0.4881	−0.1068	0.5734	0.088*	
O6	0.12843 (18)	0.00858 (12)	0.65864 (12)	0.0584 (3)	
H6	0.1101	−0.0702	0.6871	0.088*	

### Atomic displacement parameters (Å^2^)

**Table:**

	*U*^11^	*U*^22^	*U*^33^	*U*^12^	*U*^13^	*U*^23^
B1	0.0496 (11)	0.0403 (10)	0.0372 (10)	0.0098 (8)	0.0080 (8)	0.0079 (8)
B2	0.0530 (12)	0.0390 (10)	0.0376 (10)	0.0063 (8)	0.0042 (9)	0.0014 (8)
C1	0.0501 (10)	0.0455 (10)	0.0492 (10)	0.0076 (7)	0.0074 (8)	−0.0006 (8)
C2	0.0585 (11)	0.0549 (11)	0.0557 (11)	0.0042 (9)	0.0099 (9)	−0.0129 (9)
C3	0.0567 (11)	0.0713 (13)	0.0406 (10)	−0.0023 (9)	0.0063 (8)	−0.0125 (9)
C4	0.0398 (9)	0.0612 (11)	0.0362 (9)	−0.0027 (8)	0.0036 (7)	0.0018 (8)
C5	0.0307 (8)	0.0468 (9)	0.0328 (8)	−0.0008 (6)	0.0049 (6)	0.0055 (7)
C6	0.0639 (12)	0.0747 (13)	0.0333 (9)	−0.0030 (10)	−0.0013 (8)	0.0078 (9)
C7	0.0568 (11)	0.0685 (13)	0.0422 (10)	0.0011 (9)	−0.0019 (8)	0.0214 (9)
C8	0.0355 (9)	0.0518 (10)	0.0430 (9)	−0.0009 (7)	0.0045 (7)	0.0144 (7)
C9	0.0301 (8)	0.0432 (9)	0.0354 (8)	−0.0011 (6)	0.0056 (6)	0.0091 (7)
C10	0.0482 (10)	0.0480 (10)	0.0603 (12)	0.0058 (8)	0.0051 (9)	0.0234 (9)
C11	0.0540 (10)	0.0400 (9)	0.0611 (12)	0.0059 (7)	0.0121 (9)	0.0102 (8)
C12	0.0496 (10)	0.0401 (9)	0.0447 (9)	0.0034 (7)	0.0080 (8)	0.0046 (7)
N1	0.0409 (7)	0.0419 (7)	0.0380 (7)	0.0046 (6)	0.0071 (6)	0.0032 (6)
N2	0.0397 (7)	0.0395 (7)	0.0362 (7)	0.0021 (5)	0.0062 (6)	0.0064 (6)
O1	0.0702 (8)	0.0450 (6)	0.0346 (6)	0.0199 (5)	−0.0025 (5)	0.0063 (5)
O2	0.0850 (9)	0.0415 (6)	0.0373 (6)	0.0096 (6)	0.0093 (6)	0.0062 (5)
O3	0.0905 (9)	0.0455 (7)	0.0360 (6)	0.0270 (6)	0.0032 (6)	0.0077 (5)
O4	0.0538 (7)	0.0472 (7)	0.0644 (8)	0.0162 (5)	0.0151 (6)	0.0198 (6)
O5	0.0678 (8)	0.0517 (7)	0.0620 (8)	0.0202 (6)	0.0188 (6)	0.0226 (6)
O6	0.0742 (9)	0.0463 (7)	0.0606 (8)	0.0099 (6)	0.0266 (7)	0.0060 (6)

### Geometric parameters (Å, º)

**Table:**

B1—O2	1.351 (2)	C6—H6A	0.9300
B1—O1	1.355 (2)	C7—C8	1.433 (2)
B1—O3	1.361 (2)	C7—H7	0.9300
B2—O6	1.349 (2)	C8—C10	1.402 (2)
B2—O5	1.359 (2)	C8—C9	1.411 (2)
B2—O4	1.367 (2)	C9—N2	1.3612 (19)
C1—N1	1.323 (2)	C10—C11	1.358 (2)
C1—C2	1.393 (2)	C10—H10	0.9300
C1—H1A	0.9300	C11—C12	1.397 (2)
C2—C3	1.355 (3)	C11—H11	0.9300
C2—H2A	0.9300	C12—N2	1.3207 (19)
C3—C4	1.404 (2)	C12—H12	0.9300
C3—H3A	0.9300	O1—H1	0.8500
C4—C5	1.413 (2)	O2—H2	0.8501
C4—C6	1.425 (2)	O3—H3	0.8500
C5—N1	1.3559 (19)	O4—H4	0.8501
C5—C9	1.450 (2)	O5—H5	0.8501
C6—C7	1.336 (3)	O6—H6	0.8501
			
O2—B1—O1	123.27 (15)	C6—C7—H7	119.5
O2—B1—O3	116.79 (14)	C8—C7—H7	119.5
O1—B1—O3	119.94 (15)	C10—C8—C9	118.24 (15)
O6—B2—O5	121.00 (16)	C10—C8—C7	121.84 (15)
O6—B2—O4	119.75 (15)	C9—C8—C7	119.92 (16)
O5—B2—O4	119.23 (17)	N2—C9—C8	121.47 (14)
N1—C1—C2	124.43 (17)	N2—C9—C5	119.64 (13)
N1—C1—H1A	117.8	C8—C9—C5	118.88 (14)
C2—C1—H1A	117.8	C11—C10—C8	119.70 (15)
C3—C2—C1	118.03 (16)	C11—C10—H10	120.2
C3—C2—H2A	121.0	C8—C10—H10	120.2
C1—C2—H2A	121.0	C10—C11—C12	118.49 (16)
C2—C3—C4	120.11 (16)	C10—C11—H11	120.8
C2—C3—H3A	119.9	C12—C11—H11	120.8
C4—C3—H3A	119.9	N2—C12—C11	124.05 (15)
C3—C4—C5	118.01 (16)	N2—C12—H12	118.0
C3—C4—C6	121.94 (16)	C11—C12—H12	118.0
C5—C4—C6	120.05 (16)	C1—N1—C5	118.04 (13)
N1—C5—C4	121.35 (14)	C12—N2—C9	118.04 (13)
N1—C5—C9	119.78 (13)	B1—O1—H1	115.9
C4—C5—C9	118.86 (14)	B1—O2—H2	113.4
C7—C6—C4	121.23 (16)	B1—O3—H3	114.0
C7—C6—H6A	119.4	B2—O4—H4	113.0
C4—C6—H6A	119.4	B2—O5—H5	113.6
C6—C7—C8	121.04 (16)	B2—O6—H6	108.1

### Hydrogen-bond geometry (Å, º)

**Table:**

*D*—H···*A*	*D*—H	H···*A*	*D*···*A*	*D*—H···*A*
O1—H1···N2	0.85	1.90	2.7360 (16)	168.9
O2—H2···N1	0.85	1.88	2.7132 (17)	167.4
O3—H3···O1^i^	0.85	1.86	2.7076 (15)	176.8
O4—H4···O3^i^	0.85	1.89	2.7286 (16)	169.1
O5—H5···O4^ii^	0.85	1.89	2.7355 (18)	179.0
O6—H6···O2^iii^	0.85	1.95	2.7946 (17)	171.8

Symmetry codes: (i) −*x*, −*y*+1, −*z*+1; (ii) −*x*+1, −*y*, −*z*+1; (iii) *x*, *y*−1, *z*.

## References

[bb1] Clark, R. C. & Reid, J. S. (1995). *Acta Cryst.* A**51**, 887–897.

[bb2] Dolomanov, O. V., Bourhis, L. J., Gildea, R. J., Howard, J. A. K. & Puschmann, H. (2009). *J. Appl. Cryst.* **42**, 339–341.

[bb3] Lee, S. O., Kariuki, B. M. & Harris, K. D. M. (2005). *New. J. Chem* **29**, 1266–1271.

[bb4] Lehn, J. M. (1990). *Angew. Chem. Int. Ed.* **29**, 1304-1319.

[bb5] Oxford Diffraction (2011). *CrysAlis PRO* Oxford Diffraction Ltd, Yarnton, England.

[bb6] Pedireddi, V. R. (2001). *Cryst. Growth Des.* **1**, 383–385.

[bb7] Pedireddi, V. R., Chatterjee, S., Ranganathan, A. & Rao, C. N. R. (1997). *J. Am. Chem. Soc.* **119**, 10867–10868.

[bb8] Sheldrick, G. M. (2008). *Acta Cryst.* A**64**, 112–122. 10.1107/S0108767307043930 18156677

[bb9] Talwelkar, M. & Pedireddi, V. R. (2010). *Tetrahedron Lett.* **51**, 6901–6905.

[bb10] Tian, Y.-P., Duan, C.-Y., Xu, X.-X. & You, X.-Z. (1995). *Acta Cryst.* C**51**, 2309–2312.

[bb11] Westrip, S. P. (2010). *J. Appl. Cryst.* **43**, 920–925.

